# The value of renal ultrasound in children with a first episode of urinary tract infection

**DOI:** 10.4103/0256-4947.51817

**Published:** 2009

**Authors:** Layla Alshamsan, Amal Al Harbi, Khalid Fakeeh, Esam Al Banyan

**Affiliations:** From the Department of Pediatrics, King Abdulaziz Medical City, Riyadh, Saudi Arabia

## Abstract

**BACKGROUND:**

Urinary tract infection (UTI) is a common pediatric problem. Guidelines recommend obtaining a renal ultrasonogram (RUS) for young children after a first UTI. Our aim was to assess the value of routine RUS in the management of children hospitalized with a first episode of UTI.

**METHODS:**

We conducted a retrospective review of the medical records of 130 children 12 years of age or younger admitted with a first UTI. Children were excluded if they had a urinary tract abnormality before admission and/or had been treated with an antibacterial agent within 7 days before admission. The yield of RUS was measured by ability to detect renal abnormalities, by the sensitivity and specificity for detecting vesicoureteral reflux (VUR) using voiding cystourethrogram (VCUG) as a gold standard, and by its influence on UTI management.

**RESULTS:**

RUS was conducted in 130 children, but only 118 returned for a VCUG and were included in the study. The findings were positive for VUR in 20 of 40 patients (50%) with a confirmed VUR on VCUG and positive in 18 of 78 patients (23.1%) without VUR on VCUG. Of the 20 patients with a normal RUS who showed VUR, 2 had grade I reflux, 8 had grade II reflux, 7 had grade III reflux and 3 had grade IV reflux. The sensitivity, specificity, positive and negative predictive value of ultrasound in suggesting VUR was 50% and 76.9%, 52.6% and 75%, respectively. Except for one, the result of an abnormal RUS did not alter the management of our patients.

**CONCLUSION:**

The results of our study show that the RUS has a little value in the management of children with a first UTI.

Urinary tract infection (UTI) is a common pediatric problem. Guidelines from the American Academy of Pediatrics (AAP) recommend obtaining VCUG and renal ultrasonogram (RUS) for young children after a first UTI.^1^ In recent years, the clinical value of routine RUS in the management of children with a first UTI has been questioned.^2^ The aim of this study was to evaluate the yield of RUS in the management of children with first UTI.

## METHODS

All pediatric patients admitted to King Abdulaziz Medical City in Riyadh (KAMC) from 1996 to 2006 with a discharge diagnosis of UTI were identified by searching the computerized health records for UTI. KAMC is a tertiary care children's hospital in Riyadh, Saudi Arabia. The study population included all children up to 12 years of age. UTI was defined as a positive urine culture with more than 100 bacteria/mL in a midstream sample or any growth in a suprapubic bladder aspiration or in/out bladder catheterization. Children with known urinary tract abnormalities and/or who had been treated with an antibiotic 7 days prior to admission or had a history of UTI were excluded. Renal ultrasound was considered suggestive of vesicoureteral reflux (VUR) if dilatation of the pelvi-calysis, dilatation of the ureter or dilatation of collecting system of one or both kidney was reported. VCUG was performed within 1 to 6 weeks after infection. VUR was classified according to the international VUR classification.^3^ Staff radiologists read all imaging studies. The yield of RUS was measured by the ability to detect renal abnormalities, its sensitivity, specificity and positive and negative predictive value for detecting VUR, and by its impact on UTI management. The statistical analysis for determining the sensitivity, specificity, positive predictive value, negative predictive value were based on patients who had both a RUS and VCUG. Data were stored and analyzed using Epi info version 6.04d.

## RESULTS

A total of 130 patients met the inclusion criteria. Twelve patients who were booked for a voiding cystourethrogram (VCUG) as outpatients did not return for their appointment, leaving 118 patients with a first episode UTI who had VCUG performed. The mean (SD) age of the 130 patients was 21.7 months (29.8) and the median was 9 months (2 days to 132 months); 92 (68.3%) were female with a mean age of 25 months (30.6) and a median age of 12 months (2 days to 132 months) and 38 (31.7%) were male with a mean age of 11.3 months (25.9) and median age of 2 months (2 days to 120 months). All patients were symptomatic. Fever was the most common symptom. The main causative agents were *Escherichia coli* in 105 patients (80%), *Klebsiella sp* in 10 patients (7.7%), and *Pseudomonas aeruginosa* in 3 patients (2.3%). Other pathogens were responsible for 12 infections. Urine analysis was done in 129 patients and all were abnormal. Ultrasound was performed in all 130 patients with abnormal findings in 38 patients. Findings included 36 kidneys with hydronephrosis, uretric dilatation in 5 patients, dialatation of the renal calices in 2 patients and dilatation of the collecting system in 4 patients. One patient had a small renal cyst, one patient had a double collecting system, one patient had a renal scar and one patient had two small renal abscesses.

**Figure 1:**
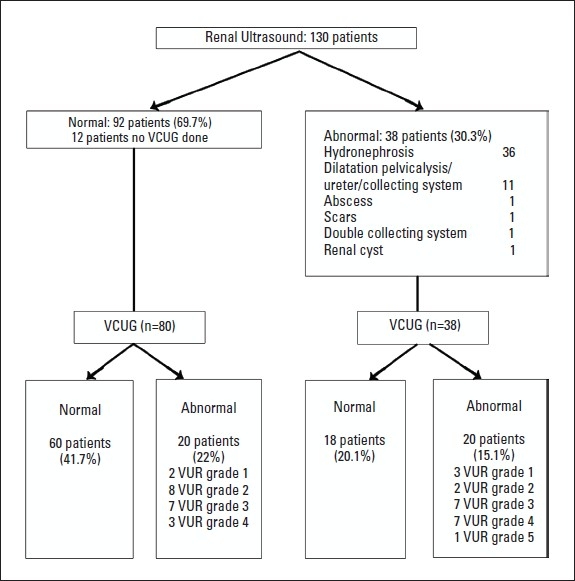
Results of imaging studies.

VCUG was performed in 118 patients. VCUG demonstrated reflux in 40 patients (Table 1). Of the 38 patients with abnormal renal ultrasound 20 patients had VUR on VCUG. The ultrasound findings of the 18 patients with no VUR included 14 patients with mild hydronephrosis, one patient with moderate hydronephrosis, one patient with moderate hydronephrosis, ureterocele, one patient with cortical scar and one patient with two small renal abscesses. Of the 92 patients with a normal renal ultrasound 20 had VUR on VCUG (Figure 1).

**Table 1:** Ultrasound results by grade of VUR on voiding cystourethogram.

VCUG Grade	Ultrasound		Total
	Abnormal	Normal	
1	3	2	5
2	2	8	10
3	7	7	14
4	7	3	10
5	1	0	1

**Total**	**20**	**20**	**40**

The sensitivity of ultrasound for detection of VUR was 50% (95% confidence interval, 36% to 68.7%), specificity was 76.9% (95% CI, 63% to 83%) the positive predictive value of ultrasound for VUR was 52.6% (95% CI, 34.1% to 65.9%) and the negative predictive value was *75% (95%* CI, 65.8% to 85.4).

In the majority of patients, RUS was performed between 2 to 8 days from admission and in some patients, it was performed after VCUG. Renal ultrasound results did not cause a change in the management of any of the children during the admission, either in antimicrobial agent or in the duration of IV therapy, with the exception of one patient with ureterocele and a double collecting system who needed referral to a urologist.

However, the course of the disease was not altered by this finding.

## DISCUSSION

The goal of imaging studies in children with UTI is to identify those at risk for renal parynchymal injury and to preserve renal function. Practice guidelines from the AAP recommend a VCUG and a renal ultrasonogram for a first UTI infection in children 2 months to 2 years of age.^1^ For older children there are no clear guidelines or consensus on the use of imaging studies. Although imaging studies are performed routinely, evidence of their value in altering management or improving outcome is limited. Only a few well-designed trials have evaluated the effect of these procedures on management and outcome. In recent years, the clinical value of routine renal ultrasound for young children in whom a first UTI is diagnosed has been questioned because of a limited effect of findings on clinical management.^2^ Our study shows that RUS findings in children with first episode UTI are of little value and did not contribute to their management except for one patient who had ureterocele and a double collecting system who needed referral to a urologist. In patients with VUR, 20 (50%) had an abnormal renal ultrasound finding which include 16 patients with hydronephrosis, 3 patients with dilatation of either the collecting system, renal pelvis or ureter, and one patient with a small renal cyst. In the remaining 20 patients with VUR, no sonographic abnormalities were found. However, the number of high-grade reflux (grade 3 to 5) was slightly higher in the first group (15 compared to 10). Several studies published have shown similar findings: Dipietro and colleagues^4^ reported 70 children, 5 years and older who underwent renal ultrasound and VCUG. Five of 70 children had an abnormal sonogram, while 2 of the 5 had reflux at VCUG. Of the 70 children, 21 had reflux, 19 (90%) of whom had no sonographic abnormality. They concluded that an abnormal sonogram does not reliably exclude VUR in children aged 5 years or older. Alon and Ganapathy studied 124 patients with UTI of whom RUS showed hydronephrosis and/or hydroureter in 10 patients. By VCUG, 38 patients were found to have VUR except for one patient, and the ultrasound findings alone had no impact on patient management.^5^ Mahant and colleague retrospectively studied children under the age of 5 years with a first episode of UTI who had renal ultrasound and VCUG. Renal ultrasound findings were suggestive of VUR in only 14 of 35 children with a confirmed VUR, and in 30 of 127 children without VUR. The sensitivity, specificity and positive and negative predictive values of ultrasound for VUR were 40%, 76%, 32% and 82%, respectively.^6^

Hoberman and colleagues conducted a prospective trial including 309 children aged 1 to 24 months, using RUS, dimercaptosuccinic acid and VCUG. Renal ultrasound results were normal in 88% and the identification of abnormalities did not modify the patient management. They conclude that renal ultrasound at the time of acute UTI is of limited value.^2^ Zamir and colleagues studied 255 children with first UTI.^7^ Thirty-three children had an ultrasound abnormality suggesting VUR, of whom only 9 had VUR on VCUG. On the other hand, in 36 children with VUR on VUG the RUS was normal. The sensitivity, specificity, positive predictive value and negative predictive value of abnormal RUS for detecting VUR were 17.7, 87.6%, 23.5% and 83.25%, respectively. These findings question the value of RUS in the management of first UTI.

Most of the published data is in children younger than 5 years of age, so we included older children in our study to add to the body of data showing that RUS findings in children with first UTI are of little value and have no influence on their management. To answer the question whether the use of routine ultrasound is justifiable in the investigation of children with a first simple UTI, several issues have to be clarified. One is the role of antenatal ultrasound as most renal anatomical abnormalities can be detected by perinatal ultrasound. Another issue is whether it is safe to omit a post-UTI renal ultrasound if the antenatal ultrasound is normal. In our study, we excluded patients with a previous diagnosis of renal anomalies so the concordance of antenatal and post-UTI renal ultrasound cannot be evaluated. The other issue is the need for ultrasound in children older than 5 years of age with UTI. Finally, the role of DMSA scan as a screening tool in children with UTI needs to be explored.

Our study has retrospective in design and including a small number of patients. In addition, there was a potential selection bias as our institution is a tertiary care hospital, but the hospital also provides primary and secondary care to the National Guard population. We tried to minimize this bias by only including patients with no urinary tract abnormalities and only those with a first UTI. In conclusion, this study suggest that ultrasound findings are neither sensitive nor specific for detecting VUR in children with a first UTI. Along with other previously published studies, it questions the value of routine renal ultrasound in the management of these children.
